# Assessing the pollution risk of soil Chromium based on loading capacity of paddy soil at a regional scale

**DOI:** 10.1038/srep18451

**Published:** 2015-12-17

**Authors:** Mingkai Qu, Weidong Li, Chuanrong Zhang, Biao Huang, Yongcun Zhao

**Affiliations:** 1 Key Laboratory of Soil Environment and Pollution Remediation, Institute of Soil Science, Chinese Academy of Sciences, 210008 Nanjing, China; 2 Department of Geography and Center for Environmental Sciences and Engineering, University of Connecticut, Storrs, Connecticut 06269.

## Abstract

The accumulation of a trace metal in rice grain is not only affected by the total concentration of the soil trace metal, but also by crop variety and related soil properties, such as soil pH, soil organic matter (SOM) and so on. However, these factors were seldom considered in previous studies on mapping the pollution risk of trace metals in paddy soil at a regional scale. In this study, the spatial nonstationary relationships between rice-Cr and a set of perceived soil properties (soil-Cr, soil pH and SOM) were explored using geographically weighted regression; and the relationships were then used for calculating the critical threshold (CT) of soil-Cr concentration that may ensure the concentration of rice-Cr being below the permissible limit. The concept of “*loading capacity*” (LC) for Cr in paddy soil was then defined as the difference between the CT and the real concentration of Cr in paddy soil, so as to map the pollution risk of soil-Cr to rice grain and assess the risk areas in Jiaxing city, China. Compared with the information of the concentration of the total soil-Cr, such results are more valuable for spatial decision making in reducing the accumulation of rice-Cr at a regional scale.

With increasing worldwide industrialization and rapid urbanization, pollution of trace metals (i.e., heavy metals) in the terrestrial environment has become a global problem^1^,^2^,^3^. Trace metals in soil may accumulate in crops, directly or indirectly threatening human health^4^,^5^. Therefore, many regulatory bodies in different countries, such as the Ministry of Health of China, the United States Environment Protection Agency (US EPA), the Food and Agriculture Organization (FAO) and Word Health Organization (WHO) of United Nations, have strictly regulated the maximum permitted concentrations of toxic trace metals in foodstuffs^6^,^7^,^8^. For Chromium (Cr), the maximum permissible limit in rice grain is 1 mg kg^−1^ in China^6^, and such standards have the force of law.

Crops can only absorb soil trace metals in dissolved (from soil solution) and exchangeable (from organic and inorganic components) forms, and trace metals in other forms cannot be absorbed directly by plants^9^. Many previous studies have shown that the availability of trace metals in soil was associated with several environmental soil factors, including pH, soil organic matter content (SOM), oxidation-reduction status (Eh), cation exchange capacity (CEC), and the concentration of calcium carbonate, clay minerals, Fe and Mn oxides^10^,^11^. Apart from the levels of trace metals themselves, soil pH plays the most important role in determining phytoavailability of trace metals in soil, due to its strong effect on speciation and solubility of trace metals both in soil and in soil solution^9^,^12^. SOM is the other major environmental factor affecting the phytoavailability of trace metals, being a major contributor to the capability of a soil to retain trace metals in exchangeable forms^9^.

The accumulation of a trace metal in rice grain is not only affected by the concentration of the trace metal in paddy soil, but also by rice variety and related soil properties. Therefore, for a specific rice variety, the degree of pollution risk of a trace metal in paddy soil is affected by multiple soil properties, such as soil pH, SOM and so on. However in previous studies, these soil factors were seldom considered in mapping the pollution risk of trace metals in paddy soil at a regional scale. Within a region, all of the environmental factors that impact trace metal phytoavailability in soil are non-stationary spatial variables, which may eventually lead to spatial non-stationarity of the soil trace metal thresholds that ensure concentrations of trace metals in crops do not exceed corresponding permissible limits. The *critical threshold* (CT) of a trace metal in paddy soil, which corresponds to the permissible limit of the trace metal in crop, may be deduced from the permissible limit and the relationships between the concentration of the trace metal in crop and its impact factors in soil. These relationships are often calibrated using traditional regression methods such as ordinary least squares regression (OLS)^13^,^14^,^15^. However, this kind of models are aspatial, that is to say, geographical location information is not considered in the estimation of model parameters^16^,^17^,^18^,^19^ and consequently the results obtained do not reflect the local characteristics of the relationships between a trace metal in a crop and its impact factors in the soil. Furthermore, traditional regression analysis methods such as OLS are based on the assumption of independence of field observations, failing to capture spatial data dependence when applied to geo-referenced data^20^. Global relationships derived from OLS regression analysis between a trace metal in rice grain and related impact factors in paddy fields may deviate considerably from those observed locally, and may provide only an average impression of the relationships over an entire region^21^. Therefore, GWR may be a useful tool to explore the spatially varying relationships between a trace metal in a crop and the impact factors in the soil. Based on the relationships and the permissible limit for the concentration of a trace metal in crop, the CT of the trace metal in soil that may ensure the concentration of the trace metal in rice grain being below the corresponding permissible limit could be deduced. The CT can reflect the sensitivity of crops to trace metals in soil. In this study, another index, *loading capacity* (LC), which is defined as the difference between the CT and the real concentration of a trace metal in soil, was assessed. The LC for a trace metal in soil reflects the risk degree of the level of the trace metal in soil to a crop.

Rice is the second most abundant crop worldwide and is the dominant grain in China^22^. Jiaxing is a typical agricultural area in east China, and rice has been planted there for a long time. Under the same soil use and management, many soil properties, such as soil pH and SOM and so on, have been in a relatively stable state in the study area. In this study, a regional scale survey in Jiaxing city, China, was carried out to map the CT of the concentration of Cr (Chromium) in paddy soil (soil-Cr) and its corresponding LC in a soil-rice system at a regional scale, under real field conditions. Soil pH and SOM are two of the major factors affecting soil processes and properties - chemical, physical and biological, such as Eh, CEC and so on^23^,^24^. Considering the redundancy among explanatory variables and the large amount of financial and human cost for other minor factors, only three major factors (i.e. soil-Cr, soil pH and soil organic matter) were adopted to explore the joint influence of soil factors on the accumulation of Cr in rice grain in the study area. The specific objectives of this study were to: (1) explore the spatially non-stationary relationships between Cr in rice grain (rice-Cr) and major related soil properties (soil-Cr, soil pH and SOM) using GWR; (2) map the spatial distribution of the CT of soil-Cr that ensures the concentration of rice-Cr not exceeding the corresponding permissible limit; (3) map the LC of soil-Cr to rice crop in Jiaxing city; and (4) delineate the risk areas where rice-Cr may exceed or may potentially exceed the permissible limit. Because the results obtained by the suggested method contained the influence of the crop variety and related soil properties, they would be valuable for developing more specific measures to reduce the accumulation of Cr in rice grain of specific variety in risk areas of the study area.

## Results and Discussions

### Sample data analysis

Selected descriptive statistics of sample data for rice-Cr concentrations and three impact factors in soil (i.e., soil-Cr, soil pH and SOM) are listed in Table 1. Rice-Cr, soil-Cr and SOM all have wide value ranges. The CVs for rice-Cr, soil-Cr, pH and SOM are 57.16%, 15.85%, 9.60%, and 35.90%, respectively, indicating that rice-Cr, soil-Cr, and SOM exhibited moderate variability in the study area while pH was of low variability. The permissible limit for the concentration of rice-Cr in Chinese National Standards is 1 mg kg^−1^ 6. In this study, five rice samples reached this level, which implied that the concentrations of soil-Cr at these five sampling points all exceeded the corresponding CTs of soil-Cr at these locations. The CTs of soil-Cr vary spatially because the accumulation of Cr in rice grain is related with many spatially variable factors, including soil-Cr, SOM content and type, and soil pH, among others, rather than just total soil-Cr.

To characterize the global infulence of the three major factors in soil on Cr accumulation in rice grain, we calculated the pearson’s non-parametric correlation coefficients between each soil property and rice-Cr (Table 2). The results show that rice-Cr is positively correlated with soil-Cr (correlation coefficient *r* = 0.71) and SOM (*r* = 0.51) and negtatively correlated with soil pH (*r* = −0.48), all being significant at the 0.01 level. These global correlation relationships indicate that soil-Cr, soil pH, and SOM all play important roles in Cr accumulation in rice grain, and soil-Cr is the most important factor among the three determinants because it has the highest correlation coefficient with rice-Cr. However, the correlation coefficients between the three soil properies are not significant at the 0.01 level. Therefore, the three major factors were adopted in exploring the joint influence on the accumulation of Cr in rice grain in the study area.

### Spatially non-stationary relationships

**Figure 1:**
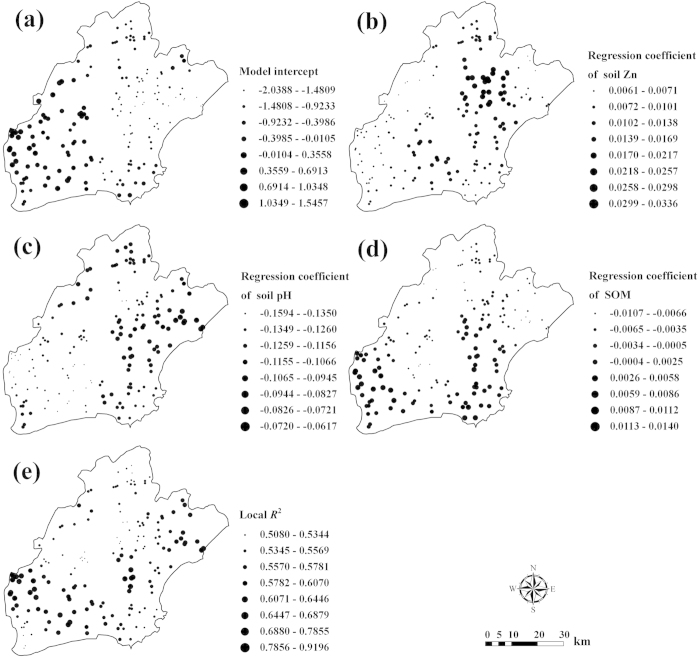
Local GWR parameters at sampling locations for Cr in rice grain against three mostly related soil properties. (**a**) model intercept, (**b**) regression coefficient of soil Cr concentration, (**c**) regression coefficient of soil pH, (**d**) regression coefficient of soil organic matter (SOM), and (**e**) local estimate of the coefficient of determination (*R*^2^) (Local GWR parameters at sampling locations were calculated using the GWR 4.0 and the maps were created using the ArcGIS 9.3).

Local regression parameters (i.e. intercept, slopes and local *R*^2^) from GWR of rice-Cr against the three explanatory variables (i.e., soil-Cr, pH, and SOM) are presented in Fig. 1. The spatially varied regression parameters estimated by GWR mean that the relationships between rice-Cr and related soil properties were spatially non-stationary, varying from one location to another over the study area (Fig. 1). The influences of the three soil properties on Cr accumulation in rice grain can be explained by their respective regression coefficients. A positive coefficient means a positive contribution while a negative coefficient indicates a negative contribution. Similarly, a large absolute coefficient value means a strong correlation while a small absolute coefficient value indicates a weak correlation. The strength of the influence of each soil factor on Cr accumulation in rice grain changes with the variation of its coefficient value over space.

The majority of soil trace metals is bound to minerals and passive SOM, and therefore are not phytoavailable^25^. However, active SOM may enhance metal phytoavailability by increasing CEC in soil, enhancing metal chelation and increasing the solubility of nutrients in soil solution^26^. Hence, the influence of SOM on trace metal accumulation in plants is not only dependent on its content, but also on its components. Moreover, the phytoavailabilities of different metal minerals vary with soil pH. However, soil pH, and the contents and speciation of soil Cr and SOM, are not the same across the study area. This fact should be the main reason that causes the effects of the three most important soil properties (i.e., soil-Cu, pH, and SOM) on the accumulation of Cr in rice grain to vary spatially (i.e., show apparent spatial non-stationarity). Rice cultivars may also have an important influence on the accumulation of Cr in rice grain^27^. In this study, the japonica rice cultivar Xiushui 63, which dominates Jiaxing city, was sampled. If other rice cultivars were planted in the study area, influence of the related soil properties in paddy fields on the Cr accumulation in rice grain might not be exactly the same^28^,^29^,^30^. Therefore, pollution risk of a trace metal in paddy soil should be closely related to the type of crops grown.

The explanatory power of the GWR model also varies spatially, as demonstrated by the local estimates of *R*^2^ for the model (see Fig. 1e). The *R*^2^ map for the GWR model has varying values from 0.51 to 0.92. Considering the redundancy among explanatory variables and the large amount of financial and human cost for other minor factors, only three major factors (i.e. soil-Cr, soil pH and soil organic matter) were adopted to explore the joint influence of soil factors on the accumulation of Cr in rice grain in the study area. It can be seen from Fig. 1e that the three major soil factors (i.e. soil-Cr, soil pH and SOM) could well explained the accumulation of Cr in rice grain. In this study, adaptive bandwidth was used in GWR when modeling the relationships between rice grain and the three most important explanatory variables (i.e., soil-Cr, pH, and SOM). Therefore, the bandwidth in the model changes spatially with the local density of sample points. As the bandwidth declines or enlarges, the GWR analysis becomes increasingly local or global, revealing more or less geographical detail like some sort of spatial microscope^20^. The sparseness of sample points in the central-northwest zone of the study area may be an important reason why the explanatory power of the GWR model is relatively weak.

### Spatial distribution of the critical threshold of Cr in paddy soil

The CT values of soil-Cr at all sampling locations were deduced using the permissible limit for rice-Cr from the Chinese National Standards and the local relationships between rice-Cr and soil properties calibrated by GWR. The mean CT value (147.28 mg kg^−1^) is far greater than the mean Cr concentration (60.63 mg kg^−1^) in paddy soil in Jiaxing city. Therefore, the risk for soil Cr exceeding its local CTs is relatively low in the study area. The CV for soil-Cr CT is 52.09%, indicating moderate variability across the study area.

**Figure 2:**
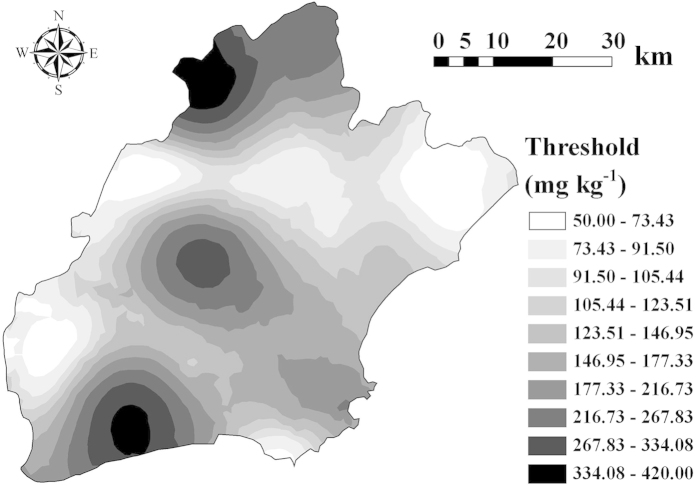
Spatial distribution of the critical threshold (CT) of Cr in soil that may ensure the concentration of Cr in rice grain being below the permissible limit of Cr in rice grain (ArcGIS 9.3).

The spatial distribution map of CT of soil-Cr, interpolated by OK, is presented in Fig. 2. Low values mainly appear in the west side and two wide east-west strips located in the middle-north and middle-south of the study area. This means that rice is more sensitive to soil-Cr in the west side and two strips than in other places in the study area.

### Assessing the risk areas of soil Cr to rice grain

**Figure 3:**
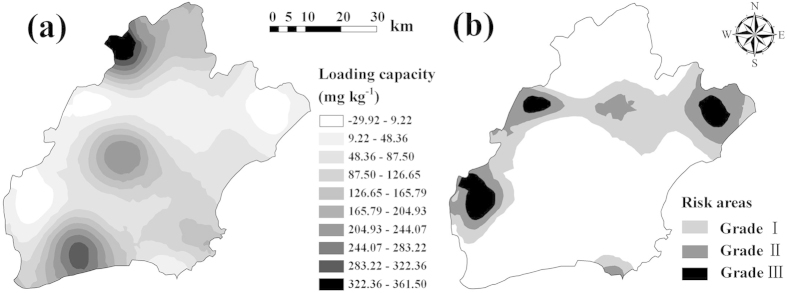
Spatial distributions of the loading capacity (LC) of Cr in soil and the risk areas. (**a**) loading capacity, (**b**) risk areas at grade I (20 mg kg^−1^ ≤ LC < 50 mg kg^−1^), grade II (0 mg kg^−1^ ≤ LC < 20 mg kg^−1^) and grade III (LC < 0 mg kg^−1^) (ArcGIS 9.3).

The LC of soil-Cr reflects the risk degree of soil Cr at a location to rice grain. The spatial distribution pattern of LC of soil-Cr (Fig. 3a) is similar to the spatial pattern of CT of soil-Cr (Fig. 2). In the study, the mean value of CT is far greater than the mean Cr concentration in paddy soil at sample locations across Jiaxing city. Thus, after soil-Cr CT values were subtracted by corresponding real concentration values of soil-Cr at each location, the overall spatial distribution trend of the differences was not much different from that of the CT values of soil-Cr across the study area. Areas with low LC values should be intensively monitored to prevent further accumulation of Cr in rice grain. The maps for risk areas delineated from the LC map based on different risk grades I, II and III (i.e., 20 mg kg^−1^ ≤ LC < 50 mg kg^−1^, 0 mg kg^-1^ ≤ LC < 20 mg kg^−1^, LC < 0 mg kg^−1^) are provided in Fig. 3b. Risk areas mainly occur in the west side and the two east-west stripes, where low LC values were observed and intensive monitoring should be first implemented in these areas. In risk areas of grade III, the LC values of soil-Cr are negative, indicating that rice-Cr may have already exceeded the permissible limit in these places. In these areas, remedial measures should be implemented, such as increasing soil pH to reduce the phytoavailability of the soil Cr, planting other crops that exhibit weaker propensity for Cr accumulation and so on. In the study, the assessment of the pollution risk of soil-Cr was based on a specific receptor, japonica rice cultivar Xiushui 63. The pollution risk of soil-Cr to another rice variety may not be the same, because the absorptive capability of rice crop to trace metals may vary with different rice cultivars.

## Materials and Methods

### Study area and data

**Figure 4:**
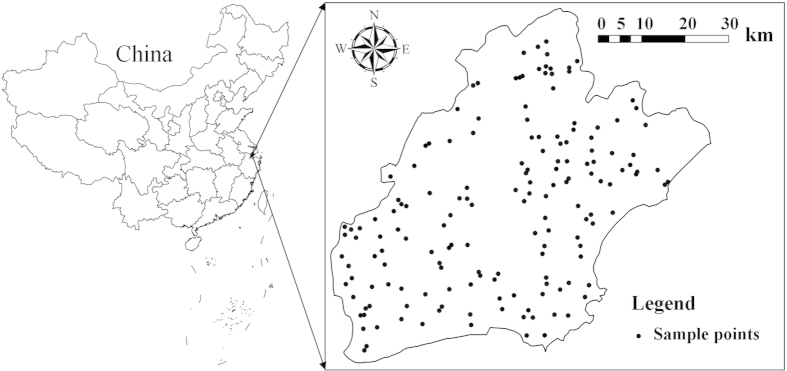
Study area and spatial distribution of sampling points (ArcGIS 9.3).

This study was conducted in Jiaxing city, a part of the Taihu Plain in northeast Zhejiang Province (Fig. 4). The study area is bounded by the longitudes of 120°18′ and 121°16′ east, and the latitudes of 30°21′ and 31°2′ north, and has an area of 3915 km^2^ (Fig. 4). Situated within the northern subtropical monsoonal climate zone, the study site has a temperate-humid climate throughout the year and four distinct seasons, with an average annual temperature of 15.9 °C and an average annual precipitation of 1168.6 mm. Due to the distribution of dense river networks and abundant water resources, Jiaxing is a typical agricultural area in east China, where paddy field is the dominant land use type. The major soil type is Stagnic Anthrosols (i.e. Gleyi-Stagnic Anthrosols, Fe-leachi-Stagnic Anthrosols, Fe-accumuli-Stagnic Anthrosols, Hapli-Stagnic Anthrosols), which were derived from lacustrine alluvium through long-term paddy cultivation^31^.

In October, 2006, random sampling of soil and rice grain was conducted in paddy fields for aquatic rice production, while mountain and urban areas were avoided. 160 pairs of samples of rice grain and surface soils (0–15 cm depth) were collected from the same locations in rice fields throughout the study area (Fig. 4). The rice genotype was mainly the japonica rice cultivar Xiushui 63. Rice plants at grain maturity (just before harvest) were cut with scissors underneath their ears. At each sampling point, 4–5 sub-samples within a distance of 10 m surrounding a specific sampling location were randomly chosen to obtain a composite sample.

Soil samples were air-dried at room temperature (20–22 °C) and after stones or other debris were removed, soil samples were sieved <2 mm. A portion of each soil sample (50 g) was ground in an agate grinder to a particle size of <0.149 mm (i.e., passing through a sieve of 1/100 mm meshes). Grain samples were oven-dried at 38 °C to constant weight. Dried rice samples were first hulled, and then ground using a stainless steel grinder into powder (<0.25 mm) for trace metal analysis. Standard methods commonly used in China were adopted to measure the concentrations of rice-Cr, soil-Cr, SOM and soil pH^32^. The total Cr concentrations of soil samples were determined using flame-atomic absorption spectroscopy (FAAS)^32^. The total Cr concentrations of rice grain samples were determined with graphite furnace atomic absorption spectroscopy (GFAAS)^32^. Soil pH was measured in a soil suspension (soil:water = 1:2.5 ) with a glass electrode^32^. Soil organic matter was obtained by the potassium bichromate wet combustion procedure^32^. For quality control, duplicates, method blanks, and standard reference materials were also analyzed.

### Geographically weighted regression

GWR was used to model the relationships between rice-Cr and three most related soil properties in paddy soil (i.e. Cr, pH and SOM). Compared with ordinary least square (OLS) regression, GWR has the advantage of taking the spatial locations of samples into consideration, permitting parameters to vary spatially, thus better reflecting the spatially varying relationships between dependent and independent variables. Here a GWR model with one dependent variable and three independent variables was re-written from Fotheringham *et al.*^20^ as:


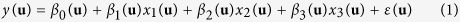


where **u** denotes a specific spatial location vector; *y*(**u**), *x*_1_(**u**), *x*_2_(**u**), *x*_3_(**u**) and *ε*(**u**) represent the dependent variable, the three independent variables (i.e. soil Cr, soil pH, SOM) and the Gaussian error term, respectively, at the spatial position **u**; *β*_0_(**u**), *β*_1_(**u**), *β*_2_(**u**), and *β*_3_(**u**) represent the model intercept for location **u** and the local regression coefficients (i.e., slopes) for independent variables *x*_1_, *x*_2_, and *x*_3_, respectively, at location **u**. The regression coefficients at location **u** can be estimated using a weighting function^20^.


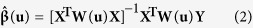


where **X** and **Y** are the one-dimensional arrays formed by the values of variables *x* and *y*; **W**(**u**) is the local weights matrix, which is calculated from a kernel function that places more weights on locations spatially closer to the calibration location^20^. As sample densities vary over space, the adaptive bi-square kernel function was adopted for weight estimation in the study. The optimal adaptive bandwidth was chosen based on Akaike information criterion as described in Fotheringham *et al.*^20^, and 35 surrounding data points were finally used for calibrating the GWR model at each location.

### Critical threshold of Cr in paddy soil

The critical threshold (CT) for the concentration of soil-Cr is defined as the maximum concentration of soil-Cr that ensures the concentration of rice-Cr is below its permissible limit. Such an index essentially reflects the sensitivity of rice grain to a trace metal in paddy soil. At a specific location, it can be deduced from the permissible limit of rice-Cr and the local relationships between rice-Cr and its impact factors in soil, which are calibrated by GWR. In this study, the CT of soil-Cr at a specific location was estimated using a function revised from Equation (1):


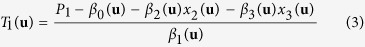


where *P*_1_ is the permissible limit for the concentration of rice-Cr, *T*_1_(**u**) is the CT of soil-Cr at location **u,** and the meanings of other symbols are the same as in equation (1). For this study, equation (3) can be more clearly rewritten as


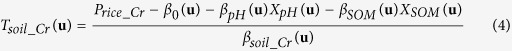


where *X* represents the value of an explanatory variable and **u** denotes a spatial location vector. In this study, *P*_*rice_Cr*_ = 1.0 mg kg^−1^.

### Kriging interpolation

In this study, ordinary kriging (OK) was used to map the spatial distributions of Cr and CT in paddy soil in Jiaxing city, based on the calculated CT values at 160 sampling locations. OK is a widely used interpolation method for continuous variables, and can minimize the variance of estimation errors and investigation costs^33^,^34^,^35^,^36^. OK is expressed as a linearly weighted average of observations in the neighborhood of unsampled location **x**_0_:


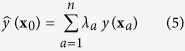


where 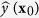 is the predicted value at location **x**_0_, 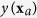 is the value of the target soil property at position **x**_*a*_, and λ_*a*_ is the weight of the corresponding datum, obtained from the ordinary kriging system with the constraint 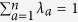. Here *n* is the number of sample data in the neighborhood. The interpolated grid had a resolution of 400 m × 400 m. Readers may see Goovaerts^37^,^38^ for more technical description of OK.

### Loading capacity of Cr in paddy soil

Here we propose the concept of “*loading capacity*” (LC): The LC for soil-Cr is defined as the difference between the CT and the real concentration of soil-Cr. This index essentially reflects the risk degree of soil-Cr to rice grain. The LC at a specific position **u** can be estimated using:


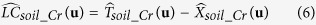


where 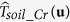 and 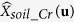 are OK estimates of the CT and the total concentration of soil-Cr at position **u**, respectively. A smaller LC value indicates higher risk for soil-Cr to exceed the CT at the position **u** and also indicates that rice grain is more vulnerable to soil-Cr. When the LC of soil-Cr at a location has a negative value, it means that rice-Cr at the location should already exceed its permissible limit.

In this study, GWR analyses and kriging interpolation were performed on a regular square grid with a cell size of 400 m × 400 m. GWR 4.0 was used for performing GWR analyses. ArcGIS 9.3 was used for geostatistical computation and producing maps.

## Additional Information

**How to cite this article**: Qu, M. *et al.* Assessing the pollution risk of soil Chromium based on loading capacity of paddy soil at a regional scale. *Sci. Rep.* **5**, 18451; doi: 10.1038/srep18451 (2015).

## Figures and Tables

**Table 1:** Sample summary statistics for Cr concentrations in rice grain and three related soil properties in paddy fields.

Properties	Min.	Max.	Mean	S.D.	C.V. (%)	Permissible limit	Numbe†
Rice Cr (mg kg^−1^)	0.00	1.15	0.46	0.26	57.16	1.00	5
Soil Cr (mg kg^−1^)	35.00	122.33	60.63	9.61	15.85		
Soil pH	4.48	8.01	6.38	0.61	9.60		
Soil organic matter (mg kg^−1^)	6.62	43.75	24.00	8.80	35.90		

^†^Number of sampling sites where rice-Cr concentrations exceed the permissible limit for rice grain.

**Table 2:** Pearson’s non-parametric correlation coefficients between rice Cr concentration and related soil properties.

Variable	Rice Cr	Soil Cr	Soil pH	Soil organic matter
Rice Cr	1	0.71**	−0.48**	0.51**
Soil Cr		1	−0.13*	0.09
Soil pH			1	0.07
Soil organic matter				1

^*^Correlation is significant at the 0.05 level (2-tailed).

^**^Correlation is significant at the 0.01 level (2-tailed).
